# The Effect of an Educational Strategy on Mothers’ Knowledge and Practices Regarding Their Children’s Oral Health

**DOI:** 10.3390/pediatric18020041

**Published:** 2026-03-12

**Authors:** Martha J. Arias-Mendoza, Emilia M. Ochoa-Acosta, Andrés A. Agudelo-Suárez

**Affiliations:** 1 Faculty of Dentistry, Santo Tomas University, Bucaramanga 681002, Colombia; 2 Faculty of Dentistry, Universidad Cooperativa de Colombia, Envigado 055422, Colombia; emilia.ochoa@campusucc.edu.co; 3 Faculty of Dentistry, University of Antioquia, Medellín 050010, Colombia

**Keywords:** health knowledge, attitudes, practice, oral health, health education, dental, health promotion, mothers, infant

## Abstract

Background/Objectives: The assessment of knowledge, attitudes, and practices (KAP) has been utilized to establish effective strategies for improving oral health in various communities. This study evaluated the effect of an educational strategy on mothers’ knowledge and practices regarding their children’s oral health. Methods: A before-and-after design was conducted in Santander, Colombia. The educational strategy was delivered through interactions with mothers via face-to-face and digital modalities. A structured questionnaire related to oral health knowledge and practices was administered before and after the educational intervention. Descriptive and paired tests were applied to observe statistically significant differences (before–after). Per-Protocol Analysis (PPA) and Intention-to-treat (ITT) analysis were performed. Ethical approval was obtained (CEBIC, 2022). Results: Fifty-eight mothers participated (median age 27 ± IQR 7 years). Observed pre–post changes were observed in the knowledge and practice dimensions, with statistically significant increases in scores and a shift from lower to higher performance categories (*p* < 0.001). Effect sizes ranged from moderate to large (*r* = 0.34–0.96), although their magnitude should be interpreted cautiously. ITT analysis showing significant changes, despite the loss of follow-up. Subgroup analyses suggested post-changes across several of the sociodemographic variables. Given the small sample size and cell counts in some categories, these analyses should be considered exploratory. Conclusions: Pre–post analyses showed changes in knowledge and practices related to children’s oral health. However, the quasi-experimental design limits causal inference and the findings should be interpreted as changes associated with the intervention. Further research and intervention alternatives are recommended from multiethnic and multicultural perspectives.

## 1. Introduction

In oral health promotion, the assessment of knowledge, attitudes, and practices (KAP) has been widely used, as it plays a fundamental role in developing strategies grounded in the social realities that communities experience daily [1,2,3]. This is a critical aspect extensively studied across different social groups and concerns various oral health conditions, such as dental caries, periodontal disease [4], oral cancer [5], and general aspects related to oral hygiene [6,7].

Analyzed from a life-course perspective, one group particularly sensitive to the implementation of epidemiological studies and/or educational strategies and intervention studies is the mother–child dyad, especially pregnant women. These women may be exposed to situations of inequity that affect their oral health and quality of life, or may experience barriers to accessing health services [8,9]. This situation is supported by epidemiological studies conducted worldwide [8,9,10,11].

Specifically in Colombia, the Fourth National Oral Health Survey (ENSAB IV) includes a dedicated analysis of pregnant women [12]. A caries prevalence of 57.5% was reported, which increases to 99.6% when initial carious lesions are considered. A secondary analysis of data from the same survey reveals that pregnant women experience inequalities in access to prenatal dental care [13]. More recent mixed-methods research highlights the role of social determinants in the reasons for dental consultations, as well as in pregnant women’s perceptions, knowledge, and practices related to oral health [14]. This situation underscores the need to continue developing health promotion and disease prevention strategies to improve these indicators and achieve better well-being among pregnant women.

Similarly, the oral health of children under two is particularly relevant in strategies targeting the mother–child dyad. According to ENSAB IV, the prevalence of caries experience at one year is 6.1% in boys and 6.0% in girls. These figures increase substantially when modified caries experience is considered (including early-stage carious lesions), reaching 33.1% in boys and 25.3% in girls [12].

The situation described above calls for the development of collective strategies across different sectors of society to improve oral health among pregnant women and in early childhood. A first step in this process involves health education as a fundamental component for promoting oral hygiene behaviors and habits, enhancing health-related knowledge, and fostering positive attitudes toward oral health and overall well-being.

International scientific literature has reported successful experiences from studies focused on parents or caregivers, as well as on the mother–child dyad, highlighting educational strategies primarily directed at mothers [15,16,17]. These approaches aim to improve oral health-related knowledge, attitudes, practices, and beliefs [18,19,20,21], which in turn may contribute to greater awareness of the importance of oral hygiene [21], improved oral health status in children, and better control of oral health risk factors [22,23].

Although numerous conceptual frameworks support educational approaches and their relationship with health promotion for the adoption of healthy behaviors and habits, the KAP model was deemed appropriate based on its scientific rationale and its capacity to address cognitive determinants and modifiable behaviors [24]. This approach may be particularly suitable for pregnant women and mothers, as oral health in early childhood is closely linked to caregivers’ actions [25]. Considering the bond established within the mother–child dyad, strategies can be implemented that leverage this relationship to promote preventive behaviors. Furthermore, this approach may be well-suited for implementation within health services, fostering comprehensive and interdisciplinary care.

Closely related to attitudes, which are examined in depth within the KAP model, are the psychosocial aspects, such as motivation, perceived responsibility, and the emotional bond that is established between mothers and their children [26,27]. These factors may contribute to adherence to oral health promotion and education programs, strengthen the sustainability of behavioral changes, and contribute to improving oral health practices [28].

Considering the importance of incorporating oral health promotion strategies for the mother–child dyad into health institution programs, this study aimed to evaluate the effect of an educational strategy on mothers’ knowledge and practices regarding their children’s oral health.

## 2. Materials and Methods

### 2.1. Description of the Study Context and Design

A broader project was designed in a city located in the department of Santander, in eastern Colombia. This project employed mixed research methods and included the assessment of mothers’ knowledge and practices related to oral health in children under two, qualitative tools such as in-depth interviews with mothers enrolled in prenatal care programs to explore their perceptions and gather insights for developing more effective educational strategies, and the evaluation of children’s oral health at 6 and 12 months following the implementation of the educational intervention with mothers. Specifically, this manuscript describes mothers’ knowledge and practices before and after the implementation of the educational strategy.

This quasi-experimental study was conducted at the “E.S.E Clínica Girón” health center located in San Juan de Girón, a historic municipality with approximately 185,000 inhabitants and a diverse ethnic composition. This municipality belongs to the department of Santander, situated in eastern Colombia near the Venezuelan border. The intervention project was drawn from a population of 184 pregnant women in the first or second trimester of pregnancy attending a maternity and paternity preparation course.

The selection of this institution and its participants was based on the opportunity to work with a population that may exhibit certain characteristics of social vulnerability, as they belong to the subsidized health insurance regime within the national health system. This means that women were individuals without the financial capacity to pay for health insurance (as employed populations do) and who receive care within the state-funded public health system.

This context provided an opportunity to incorporate oral health education strategies into the broader health actions directed toward mothers during pregnancy and throughout the child’s early years.

### 2.2. Study Participants

**Figure 1:**
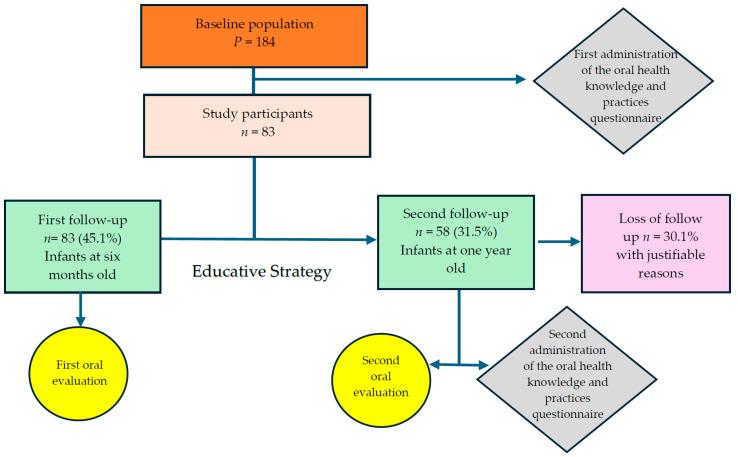
Flowchart for the study design and the sampling process.

At the first follow-up, conducted when the infants were six months old, 83 mothers participated. Following the implementation of the educational intervention, in a second follow-up conducted when the children were approximately one year of age, 58 mothers completed the assessment. Loss to follow-up occurred due to several reasons, including changes in health insurance status from the subsidized to the contributory system, changes in residential zoning, attrition among migrant participants, loss of contact information, and family-related circumstances such as children being placed under the care of the Colombian Institute of Family Welfare (ICBF) or caregivers relocating to other cities. Figure 1 shows the flowchart of the sampling process including all the characteristics of the macro-project.

### 2.3. Educative Strategy

The educational strategy titled “In Tune: Mother and Child, a Bond of Love” (“En sintonía: madre e hij@, un lazo de amor” in Spanish) was developed through sustained interaction with mothers, drawing on the collection of existing knowledge and practices concerning oral health, participant observation, and the identification of communicative and educational barriers at different stages of the maternity and paternity preparation courses conducted at the E.S.E. Clínica de Girón.

This approach is aligned with a public health perspective, considering the comprehensive application of multiple methods that may generate greater adherence, engagement, and motivation within the field of oral health promotion, based on the theoretical frameworks used by professionals working in primary health care services [27].

The strategy addressed five thematic areas and was implemented through both face-to-face and digital modalities. Digital materials were shared via WhatsApp, while in-person sessions with pregnant women were used to reinforce key topics. The themes covered included (1) meanings attributed to the mouth and oral health, linked to specific hygiene practices such as tooth brushing; (2) maternal nutrition during pregnancy, emphasizing the importance of food preferences transmitted to the baby for improving overall health; (3) breastfeeding, considered a meaningful and significant practice for mothers; (4) infant oral hygiene, focusing on how, when, how often, and with what tools oral cleaning should be performed; (5) caregiving practices related to complementary feeding and maxillary development.

Mothers received oral health educational materials, guidance on accessing dental care within Colombia’s national General System of Social Security in Health, and an oral hygiene kit (toothbrush, toothpaste, and dental floss). In addition, after the babies’ birth they attended two in-person educational follow-up sessions at 6 and 12 months and were provided with age-appropriate oral hygiene tools for their children (finger toothbrushes and infant toothbrushes). Children underwent comprehensive dental examinations, and when indicated, fluoride varnish was applied to early carious lesions. These findings will be explained in subsequent publications of the research team.

Fieldwork was conducted between January 2024 and October 2025.

### 2.4. Questionnaire Description and Study Variables

A structured questionnaire in Spanish was developed by an interdisciplinary research team with expertise in pediatric dentistry, public health, and epidemiology, using both qualitative and quantitative methodological approaches. For the design of the instrument, an extensive review of the scientific evidence was conducted, along with pilot testing in the target population, followed by a rigorous process of face and content validation with experts, calculating content validity indices. The details of this process are pending publication (in press). The questionnaire comprises four sections: (1) sociodemographic characteristics (9 items), including age, ethnicity, socioeconomic status, marital status, educational level, number of children, occupation, and place of residence; (2) psychosocial dimension (7 items), which focuses on maternal attitudes and motivations, as well as on emotional aspects and the bond established between the mother–child dyad in oral health education; (3) oral health knowledge (13 items), assessing maternal knowledge during the first year of life; (4) oral health practices (11 items), exploring maternal behaviors related to the promotion and maintenance of oral health in infants.

The initial version of the questionnaire was administered once to a sample of 269 women from two cities in eastern Colombia; methodological details have been reported elsewhere [29]. Internal consistency of the response scale was acceptable, with a Cronbach’s alpha of 0.774 and a McDonald’s omega of 0.758. Instrument reliability and reproducibility were further evaluated using a test–retest procedure in a subsample of 30 women, yielding an intraclass correlation coefficient (ICC) of 0.702 (95% CI: 0.374–0.858), indicating moderate reliability.

The main study variables (dependent variables) were grouped into three dimensions (as proposed by the questionnaire): psychosocial (D-items), oral health knowledge (K-items), and oral health practices (P-items). For each dimension, items were originally measured using Likert-type response scales and analyzed descriptively. Subsequently, responses were dichotomized as follows: negative/positive (D), incorrect/correct (K), and inadequate/adequate (P). Positive, correct, and adequate responses were summed to generate dimension-specific scores as well as an overall score.

Raw scores were then divided by the maximum possible score and multiplied by 100, yielding standardized scores ranging from 0 to 100. Based on consensus among the research team, standardized scores were classified into three overall levels for each dimension—Low (0–59), Good (60–79), and Excellent (80–100)—enabling the data to be examined using multiple statistical analytical approaches.

The cut-off points used to define these ordinal levels were established by the research team through theoretical discussion grounded in the conceptual framework of the study (KAP model) and considering an educational intent. This categorization aimed to reflect meaningful transitions in knowledge and practice levels rather than purely statistical distributional criteria. Both continuous scores and qualitative levels were retained for integrative purposes; continuous measures permit an initial analytic approach, and categorical levels were used for interpretative purposes, and to facilitate a public health understanding of the observed changes, to be aligned with the educational objectives of the intervention.

### 2.5. Statistical Analysis

First, a descriptive analysis of the study’s sociodemographic variables was performed, along with the percentage distribution of the items for each dimension at both time points (before and after the educational intervention), using categorical and dichotomous classifications. Normality tests were then applied to the accuracy scores for each dimension, supporting the use of non-parametric analyses. Accordingly, differences between pre- and post-intervention scores were assessed using the Wilcoxon signed-rank test, and effect sizes were calculated using rank-biserial correlation (≤0.29: small; 0.30–0.49: moderate; ≥0.50: large).

Subsequently, based on ordinal categorical levels defined for each dimension, paired contingency analyses were conducted between the two time points. Changes in paired categorical variables with three levels were evaluated using Bowker’s test of symmetry, an extension of McNemar’s test for k × k contingency tables. These paired analyses with the three categories (Low, Good, Excellent) enable the calculation of two independent indicators. Gross mobility (GM) was calculated as the proportion of paired observations falling outside the main diagonal of the square contingency table. Net directional change (NDC) was computed as the difference between upward (Low → Good/Excellent; Good → Excellent) and downward (Good → Low; Excellent → Good/Low) transitions divided by the total number of valid pairs.

The scores and the ordinal categorical levels for each dimension were analyzed by subgroups, considering the sociodemographic variables included in the study.

It is important to mention that these analyses correspond to a Per-Protocol Analysis or Completers Analysis (PPA). A comparison was conducted between the scores obtained at Moment 1 and the original categorical levels for both the 58 women who continued with the educational strategy (G1) and the 25 who withdrew (G2). Non-parametric (Mann–Whitney U) tests and chi-square analyses were performed to verify the absence of statistically significant differences between the two groups and to assess the adequacy of the analytical approach used.

However, considering that this study did not include a control group and experienced substantial attrition (albeit for justified reasons), an intention-to-treat (ITT) analysis was conducted, which involved the inclusion of all 83 women who initially entered the study [30]. The baseline observation carried forward (BOCF) method was applied [31], whereby the 25 participants who withdrew were assigned their Moment 1 scores, assuming a conservative worst-case scenario in which their knowledge and practices did not change following the educational intervention. This method was considered appropriate in the context of this type of study, because of the loss of information was justified.

Finally, a bivariate analysis was conducted to compare the scores obtained at moment 2 according to sociodemographic variables. Given the non-parametric nature of the data, non-parametric tests were applied. Mann–Whitney U tests were used for dichotomous sociodemographic variables, while Kruskal–Wallis tests were applied for polytomous sociodemographic variables.

Statistical significance was set at *p* < 0.05. SPSS software version 29.0-IBM^®^ (NY, USA) and Jamovi 2.6.44 (open statistical software, Australia) were used for all analyses.

### 2.6. Ethics

The study was conducted per the Declaration of Helsinki principles as the ethical framework for research involving human subjects [32]. All participants provided written informed consent after being fully informed about the study objectives and their potential implications. This research complied with national regulations governing research involving human subjects (Resolution 8430 issued by the Ministry of Health), under which it is classified as a risk-free study [33]. It adhered to international ethical standards established by the Council for International Organizations of Medical Sciences (CIOMS) [34]. The study protocol was reviewed and approved by the Committee on Ethics, Bioethics, and Scientific Integrity (CEBIC, Spanish acronym) of Santo Tomás University (Act No. 28, 13 December 2022), Bucaramanga, Colombia.

Taking into account the researchers’ ethical commitment to the study participants, public health actions included within the broader macro-project were incorporated and warrant special attention. The educational strategy described these actions.

Finally, the preparation of this manuscript followed the TREND statement for nonrandomized evaluations, as well as the TIDieR-PHP checklist and guide for the description of the educational intervention, both adapted for behavioral and public health interventions [35,36].

## 3. Results

### 3.1. Sociodemographic Characteristics of the Participants

Table 1 summarizes the participants’ sociodemographic characteristics according to the study completion. Regarding the participants who completed the study (*n* = 58), the median age was 26 (± 7) for women who completed the study. Nearly two-thirds were married, or cohabiting, and over 60% had completed secondary education. Slightly more than half were primiparous, and 88% reported being homemakers. Most participants belonged to the low socioeconomic strata (97%). Venezuelan nationality was reported by 14%, and 2% (*n* = 1) identified as belonging to an ethnic minority (Indigenous). With regard to the participants who were lost to follow-up (*n* = 25), baseline characteristics and data trends were similar to those of the participants who continued to moment 2 of the study following the educational intervention.

### 3.2. Characteristics of the Core Questionnaire

The Supplementary Materials (Tables S1–S6) provides detailed information on the questions formulated for each dimension of the instrument for participants who completed the study. It also presents the different response options for the questionnaire, including polytomous responses (e.g., agreement or decision scales) and dichotomous responses (e.g., positive/negative, correct/incorrect, adequate/inadequate). This information enables a clearer understanding of how responses were distributed at both time points of the study, facilitating a more accurate categorization of the phenomenon of interest.

**Figure 2:**
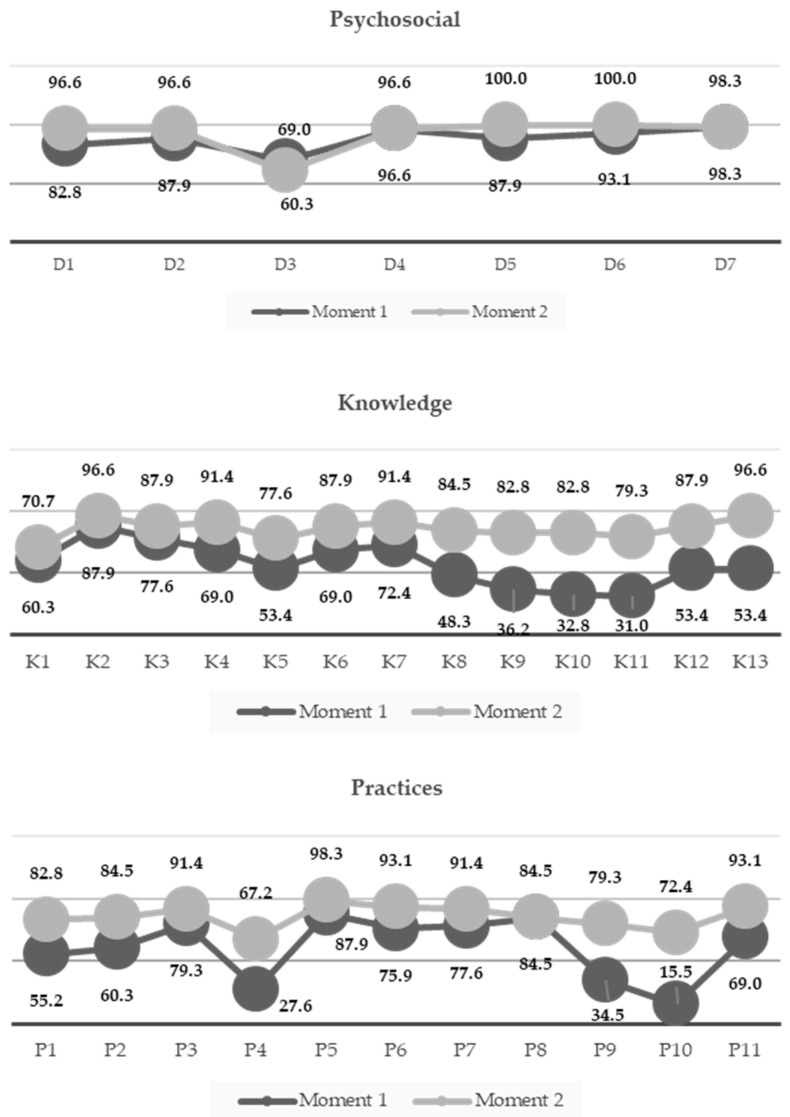
Percentage of correct responses of the core questionnaire before and after the educative strategy. Santander, Colombia, 2025 (*n* = 58).

Figure 2 shows the percentage of responses categorized as correct for each question, according to the dimensions analyzed in the study and across the two time points (moments 1 and 2: before and after implementation of the educational strategy). Regarding the psychosocial dimension, high ceiling effects were observed at moment 1 (values ≥ 83% of participants with positive responses), except for item D3. Although higher percentages were observed in a large proportion of the items at moment 2—reaching values of 97% or more—no positive changes were observed in dimension D4, and, unexpectedly, a decrease in the percentage of positive responses was observed for item D3.

For the knowledge dimension, an overall upward trend in the percentage of correct responses was observed, with particularly marked increases for questions K9, K10, and K11, while less pronounced increases were noted for questions K1 and K2. Concerning the practices dimension, at moment 2, the percentage of correct responses ranged from 83% to 93%, with the most notable increases between the two time points observed for questions P4, P9, and P10 (Figure 2).

Considering that the questionnaire allowed the calculation of standardized scores ranging from 0 to 100 for each dimension, baseline values for dimensions D, K, and P were compared between participants who completed the study and those who withdrew. The following results were obtained: 1) D: *p* = 0.940; 2) K: *p* = 0.743; and 3) P: *p* = 0.901. These findings indicate that baseline characteristics did not differ substantially between groups at the start of the study in terms of oral health knowledge and practices, thereby supporting the appropriateness of conducting the per-protocol analysis (PPA).

### 3.3. Effect of the Educative Strategy

Table 2 presents the scores obtained at both time points, including measures of central tendency and dispersion for each dimension and for the overall instrument. Overall, changes were observed across all dimensions, reflected in increased scores. Statistically significant differences were found for the knowledge and practices dimensions (*p* < 0.001), while a p-value of 0.08 was observed for the psychosocial dimension, suggesting a borderline association (*p* < 0.10). Effect sizes ranged from moderate to large (*r* = 0.34–0.96); however, these values should be interpreted with caution given the study design.

Considering the losses to follow-up during the implementation of the educational strategy, ITT analyses were performed using the BOCF approach described in the Methods section. The following test statistics were obtained: 1) D: *p* = 0.083; 2) K: *p* < 0.001; and 3) P: *p* < 0.001. Effect sizes were calculated as follows: 1) D = 0.341; 2) K = 0.923; and 3) P = 0.651. These results indicate that, even under the conservative worst-case scenario assumption—namely, that participants who withdrew experienced no improvement in knowledge and practices—observed post changes were maintained in the target population (with significant statistical differences).

Table 3 shows the paired contingency analyses. These analyses revealed statistically significant associations across all evaluated domains (*p* ≤ 0.012 for all comparisons). Participants who were initially classified as having low levels in the psychosocial, knowledge, or oral health practices dimensions achieved good or excellent levels at moment 2. For instance, two-thirds or more of the participants who initially presented low levels of oral health knowledge and practices reached excellent levels in the same dimensions at moment 2 (*p* < 0.001). This indicates that the post-intervention changes in scores across the dimensions were also reflected in a qualitative increase in the ordinal levels of knowledge and practices among the participants regarding their children’s oral health. However, another group of participants remained in the same category, albeit in a smaller proportion.

The magnitude of change varied by dimension, with very high mobility observed for Knowledge (GM = 0.81; NDC = +0.71). All observed changes were directionally positive, indicating consistent shifts toward higher categories after the educational intervention. The comparatively lower mobility observed in the psychosocial dimension may be partially explained by ceiling effects, given the high proportion of participants already classified in the highest category at baseline.

The results observed in the contingency tables were maintained when the ITT analysis was performed, including participants considered lost to follow-up. The analyses revealed statistically significant improvements across all evaluated domains (*p* ≤ 0.012 for all comparisons), even under the conservative worst-case scenario assumption for those who withdrew, with consistent trends observed in the Gross Mobility (GM) and Net Directional Change (NDC) indicators.

### 3.4. Analysis of the Effect of the Educational Strategy by Subgroups

Considering the characteristics of the study and the implications of its design, an analysis of score changes across the different dimensions involved in the educational strategy was conducted by subgroups (according to sociodemographic variables). The significance of the differences between moment 1 and moment 2 is shown in Table 4.

When analyzing the psychosocial dimension (D), statistically significant differences (indicating increased scores) were observed only among women aged 27 years or older. Regarding the K and P dimensions, increased scores were observed following the implementation of the educational strategy across a substantial proportion of the sociodemographic variables considered, except for participants with technical or technological education, those who were employed, self-employed, or students, those from middle socioeconomic strata, and those belonging to minority ethnic groups (Indigenous). However, given the relatively small sample size and small cell counts in some categories, these analyses should be interpreted as exploratory.

### 3.5. Additional Analyses

Additional analyses are provided by Supplementary Materials (Tables S7 and S8). This tables offers information about the variables related to mothers’ knowledge and practices in oral health after the implementation of the educational strategy.

Table S7 presents the scores obtained at moment 2 for each dimension, according to the study’s sociodemographic characteristics. Statistically significant differences in the scores were identified only in the knowledge and practices dimensions according to educational level, and in the practices dimension according to ethnicity.

Table S8 displays the relative frequencies of mothers obtaining excellent levels in the different dimensions according to the sociodemographic variables. Statistically significant differences in the percentual distribution were identified in K and P dimensions (separately and together) when education is considered (women with Technical-Technological education reported more frequently excellent K levels, and those with secondary education reported excellent P levels). For the other variables, no statistically significant differences were observed (*p* > 0.05).

## 4. Discussion

### 4.1. Main Study Findings

The study’s main findings suggest observed changes between the pre- and post-intervention assessments in mothers’ knowledge and practices regarding the oral health of children under two years of age. When the dimensions were analyzed, improvements were observed, reflected in higher scores corresponding to good or excellent levels, with statistically significant differences in scores, except when the psychosocial dimension was analyzed as a continuous (quantitative) variable. Moderate to large effect sizes were observed; however, these results should be interpreted with caution.

These findings persist despite the losses to follow-up, as the intention-to-treat (ITT) analysis demonstrated significant changes in maternal knowledge and practices after the educational intervention. Subgroup analyses showed post-changes among across analyzed sociodemographic variables. However, these analyses should be interpreted with caution given the small sample size and limited cell counts in some categories. Considering prior evidence from the literature, it is worth highlighting that, to the best of our knowledge, this is one of the few studies published in Colombia that addresses this topic using this educational strategy.

### 4.2. Possible Explanations for the Study Findings

One aspect of particular interest in this study concerns the psychosocial dimension. Although this dimension has traditionally been associated with the attitudinal component of the KAP model in health research, our study incorporated items related to the mother–child bond. This bond may foster more positive maternal behaviors, perceptions, and motivations regarding their children’s oral hygiene, promote the recognition of oral diseases as important issues in overall health, and reinforce breastfeeding as a key factor supporting optimal craniofacial growth and development—qualities essential for good oral health [37,38,39].

In our study, the psychosocial dimension showed smaller changes compared to the other dimensions. Baseline scores were already high, creating ceiling effects. This should not be considered a negative finding; on the contrary, it may be related to the fact that for many mothers this was their first child and the mother–child bond was already strong at the beginning of the intervention.

From a health promotion perspective, having a high level of bonding and positive attitudes toward health facilitates greater adherence to health promotion and prevention programs at both institutional and community levels.

Several psychological theoretical models—such as attachment theory [40], the theory of planned behavior [41], and social cognitive theory [42]—can help explain how the bond developed between mother and child, along with maternal self-efficacy, contributes to positive attitudes, motivations, and perceived behavioral control. These factors, in turn, may lead to improvements in practices related to healthy feeding, hygiene, oral health, and the utilization of dental care services.

For instance, a Japanese study showed that disturbances in the mother–child affective bond can negatively influence the quality of children’s oral hygiene, particularly tooth brushing practices [43]. Another study in Bangladesh, applying the theory of planned behavior, found that inadequate maternal preventive behaviors were associated with a higher prevalence of early childhood caries [38]. Finally, research in Iran demonstrated that maternal self-efficacy is a significant predictor for the prevention of primary tooth caries among children [44].

When oral health knowledge and practices were analyzed, the results demonstrated changes after the implementation of the educational strategy among the participating mothers. These changes persist despite loss to follow-up, a finding confirmed by the ITT analysis. The scientific literature has reported randomized controlled trials and community-based studies that demonstrated successful experiences regarding the role of educational and intervention strategies in improving knowledge and practices related to oral hygiene and oral health in general. Although our study used an uncontrolled design, these findings provide a contextual framework that may help interpret our results [45,46].

Research describes a wide variety of educational and intervention strategies implemented among pregnant women and mothers of children under two, including home visits, information and communication technology (ICT)—mediated strategies, motivational interviewing, and telephone follow-up, among others [24,45,46,47].

Complementing the above, the educational strategy implemented in the Colombian setting was delivered through educational materials sent via WhatsApp and reinforced by face-to-face activities. The role of social media in promoting health education is undeniable, and according to the literature, several studies have demonstrated the effectiveness of WhatsApp-delivered messages in improving oral health knowledge and promoting adequate oral hygiene practices among pregnant women and children [48,49]. In Colombia, according to the most recent National Demographic and Health Survey (ENDS 2025), 93% of households report having a mobile phone as a consumer good [50]. This is particularly relevant, as it highlights the feasibility of implementing educational strategies through social media platforms.

However, future research must compare different types of educational approaches or strategies—both face-to-face and those delivered through remote means and social media—using comparative intervention study designs. This would provide stronger evidence regarding their effects on oral health knowledge and practices across different settings and populations.

The subgroup analysis revealed statistically significant changes in maternal knowledge and practices regarding their children’s oral health across several sociodemographic variables. The integrative nature of the educational intervention may partly explain these findings. However, it is important to note that not all variables were sufficiently represented in absolute numbers, which may have influenced the statistical analyses and supported the interpretation of these results as exploratory.

Variables such as migratory status and ethnicity highlight the need for further research and intervention strategies using innovative methodologies that adopt a cultural perspective, recognizing local knowledge systems and diverse worldviews [51,52].

Although minority ethnic groups were not highly represented in absolute or relative frequencies in this study, this variable should be considered in research studies and in public health and oral health promotion interventions. According to the National Administrative Department of Statistics of Colombia (DANE) and data from the 2018 National Population and Housing Census, approximately 12% of the Colombian population identifies as belonging to a minority ethnic group, with 4.4% identifying as Indigenous, representing just over 1.9 million people [53].

Regarding migration, data from the Inter-Agency Coordination Platform for Refugees and Migrants from Venezuela (R4V) indicated that by the end of 2024, the Venezuelan population in Colombia was approximately 2.8 million [54]. This underscores the need for public health research to consider migration as a key analytical variable, a consideration that is equally relevant in oral health research [55]. In our study, there was representation of mothers from a neighboring country (although lower than might be expected), and subgroup analyses showed statistically significant changes in their knowledge and practices.

Finally, it is important to note that the educational strategy included several components (educational materials, oral hygiene kits, clinical examinations, and preventive measures such as fluoride varnish). Therefore, the observed behavioral changes may reflect the combined effect of multiple intervention components. From a public health perspective, strategies targeting the mother–child dyad should be comprehensive and allow the adoption of multiple tools, each with specific objectives and goals that contribute to achieving desired changes in the population. Such factors may interact positively with strategies identified in the literature as effective in oral health promotion and prevention programs [15,16,17,18,19,20,21,22,23].

### 4.3. Scope and Limitations of This Study

One of the strengths of this study is the careful design of the educational strategy, which incorporated multiple quantitative and qualitative data collection tools and drew on diverse information sources. This approach achieved high levels of acceptability and engagement among participating mothers and enabled the implementation of additional intervention activities in the mother–child dyad. The data collection instrument assessing knowledge and practices was also carefully developed and reviewed by subject-matter experts; however, it will be reported separately in full (data not yet published). Although the sample size was relatively small, statistically significant differences were observed in pre–post changes, not only when analyzed overall, but also when examined by subgroups and using both analytical approaches (PPA and ITT), showing statistical sensitivity to detect meaningful changes after the educational intervention.

Nevertheless, several important limitations should be considered when interpreting the findings. The absence of an external control group limits causal inference. It cannot be ruled out that oral health knowledge and practices are highly sensitive to external influences, including social media, mass communication channels, and other informal information sources. While such sources may be beneficial in some cases, they also contribute to misinformation regarding oral hygiene and oral health. In this context, the role of educational agents is crucial to guide, verify, and provide feedback on existing knowledge and practices, thereby supporting the effectiveness of oral health promotion strategies.

We recognize that a considerable proportion of attrition occurred from baseline to final follow-up. However, the reasons for loss to follow-up were beyond the direct control of the research team and were likely influenced by contextual and social factors related to the study setting and the characteristics of participating mothers. Taking this into account, the research team conducted the corresponding analyses considering loss to follow-up (ITT) to verify that, even under the worst-case scenario, positive and statistically significant changes were maintained after the educational intervention.

With regard to the results found in the statistical analysis (effect sizes), values very close to 1 were observed, which may initially seem unusual for studies related to human behavior. However, this aspect should be interpreted from both methodological and conceptual perspectives.

To mitigate potential biases related to score compression, dichotomization, and attrition, the ITT analyses retained the effect sizes obtained for the scores, demonstrating positive changes following the educational intervention. Another analytical element relates to the ceiling effects observed in the psychosocial dimension, which has been explained in detail elsewhere.

Unlike other behavioral studies in the psychological field, this study evaluated two constructs or dimensions that, within oral health education, aim to improve very basic knowledge and practices in oral health—domains that typically exhibit steep learning curves, reflected in the observed changes when variables are analyzed either quantitatively (scores) or qualitatively (levels). Although the lack of a control group limits internal validity, the decision was guided by ethical considerations, as withholding an intervention with potential health benefits for mothers and children was not deemed appropriate in this context.

It is also important to consider, when interpreting the results, that although positive changes were observed following the educational strategy, other factors may have contributed to the outcomes reported in this study. These may include repeated testing effects, social desirability bias, and concurrent healthcare contact.

Despite these limitations, this study provides valuable preliminary evidence on the effectiveness of educational interventions aimed at improving mothers’ oral health knowledge and practices related to infant oral health in real-world settings. Further research should explore additional educational strategies applicable to other specific contexts and should consider the use of qualitative, mixed-methods, and participatory action research approaches to enhance community engagement and empowerment.

Another aspect is that the educational strategy was implemented mainly with mothers, many of whom were homemakers, in a specific cultural and social context. Future studies should involve fathers and other significant adults, which may increase the intervention complexity and require adaptation of the questionnaires to additional key actors.

Although the strategy also includes dental examinations in children and other methodological techniques (they will be shown in subsequent publications by the research group), ensuring continuity and short- and medium-term follow-up of mothers and children is essential. As children enter early education settings, complementary strategies involving caregivers and health professionals should also be considered.

The broader project from which this quasi-experimental study is derived should continue contributing to the democratization of knowledge by aligning research processes with actions that enhance community quality of life through engagement with local, cultural, and experiential knowledge beyond the academic sphere [56]. In this regard, it is important for intervention strategies to build institutional capacity in health services, thereby enabling the establishment of sustainable programs with the participation of different health professionals. This was one of the project’s objectives.

Within the framework of Colombia’s science, technology, and innovation system, a document on the social appropriation of knowledge—a nationally recognized concept, also referred to in other contexts as knowledge translation, knowledge mobilization, or knowledge transfer—was developed for consideration by the health institution where the research was conducted. This document may also serve as a model for replication in other health institutions.

## 5. Conclusions

The pre–post analyses showed improvements in the knowledge and practices of pregnant women and mothers related to their children’s oral health after the implementation of the educational strategy. This effect was reflected in increased scores across each dimension and overall, as well as in improvements in qualitative levels. However, given the quasi-experimental design, caution is required when interpreting the findings in causal terms. Further research and intervention alternatives are recommended from multiethnic and multicultural perspectives, along with short-, medium-, and long-term follow-up of actions, including the assessment of infants’ oral health status.

## Figures and Tables

**Table 1:** Baseline sociodemographic characteristics of participants according to study completion status. Santander, Colombia, 2025 (*n* = 83).

Variables	Completed		Lost to Follow-Up	
	*n*	%	*n*	%
Age *				
Median (IQR; Min–Max)	25	7; 16–41	26	4; 17–35
Age categorized				
≤26	38	65.5	18	72.0
≥27	20	34.5	7	28.0
Marital status				
Single	19	32.8	6	24.0
Married/Cohabiting	39	67.2	19	76.0
Education				
≤Primary	19	32.8	7	28.0
Secondary	35	60.3	15	60.0
Technical-Technological	4	6.9	3	12.0
Number of children				
0 (Primigravida)	33	56.9	10	40.0
One	17	29.3	12	48.0
Two and more	8	13.8	3	12.0
Occupation				
Employed	3	5.2	1	4.0
Housewife	51	87.9	20	80.0
Independent	3	5.2	2	8.0
Student	1	1.7	2	8.0
Socioeconomic stratum				
Low (1–2)	56	96.6	24	96.0
Medium (3–4)	2	3.4	1	4.0
Origin Country				
Colombia	50	86.2	20	80.0
Venezuela	8	13.8	5	20.0
Ethnicity				
No	1	1.7	1	4.0
Indigenous	57	98.3	24	96.0
All	58	100.0	25	100.0

* Normality tests were carried out. IQR: Interquartile Range.

**Table 2:** Scores obtained by participants who completed the study across the different dimensions before and after the educational strategy. Santander, Colombia, 2025 (*n* = 58).

Main Scores *	Moment 1						Moment 2						*p*-Value **	Effect Size ***
	$\overset{-}{X}$	SD	Me	IQR	Min	Max	$\overset{-}{X}$	SD	Me	IQR	Min	Max		
D	87.9	16.7	100.0	14.3	28.6	100.0	92.6	10.1	100.0	14.3	42.9	100.0	0.083	0.341
K	57.3	19.9	53.8	30.8	15.4	100.0	86.2	14.9	92.3	23.1	30.8	100.0	<0.001	0.923
P	60.7	18.7	54.5	27.3	27.3	100.0	85.3	12.4	90.9	27.3	54.6	100.0	<0.001	0.960

$\overset{-}{X}$: Mean; SD: standard deviation; Me: Median; Min: Minimum; Max: Maximum; * Normality tests were carried out; ** Nonparametric Wilcoxon signed-rank test; *** Rosenthal’s r.

**Table 3:** Paired comparison across the different dimensions before and after the educational strategy for participants who completed the study. Santander, Colombia, 2025 (*n* = 58).

Paired Contingency Variables							
D- Moment 1	D- Moment 2			Total *n* (%)	*p*-value *	GM **	NDC (+) **
	Low *n* (%)	Good *n* (%)	Excellent *n* (%)				
Low	1 (20.0)	1 (20.0)	3 (60.0)	5 (5.4)	0.012	0.19	0.19
Good	0 (0.0)	0 (0.0)	7 (100.0)	7 (12.5)			
Excellent	0 (0.0)	0 (0.0)	46 (100.0)	46 (82.1)			
Total	1 (1.7)	1 (1.7)	56 (96.6)	58 (100.0)			
K- Moment 1	K- Moment 2			Total *n* (%)	*p*-value *	GM **	NDC (+) **
	Low *n* (%)	Good *n* (%)	Excellent *n* (%)				
Low	2 (6.7)	8 (26.7)	20 (66.7)	30 (51.7)	<0.001	0.81	0.71
Good	1 (4.8)	4 (19.0)	16 (76.2)	21 (36.2)			
Excellent	1 (14.3)	1 (14.3)	5 (71.4)	7 (12.1)			
Total	4 (6.9)	13 (22.4)	41 (70.7)	58 (100.0)			
P- Moment 1	P- Moment 2			Total *n* (%)	*p*-value *	GM **	NDC (+) **
	Low *n* (%)	Good *n* (%)	Excellent *n* (%)				
Low	2 (6.3)	9 (28.1)	21 (65.6)	32 (55.2)	<0.001	0.67	0.64
Good	0 (0.0)	5 (38.5)	8 (61.5)	13 (22.4)			
Excellent	0 (0.0)	1 (7.7)	12 (92.3)	13 (22.4)			
Total	2 (3.4)	15 (25.9)	41 (70.7)	58 (100.0)			

* McNemar–Bowker test for paired contingency tables with more than two categories. ** GM (Gross mobility) ranges from 0 to 1 and reflects the overall magnitude of categorical change. NDC (Net directional change) ranges from −1 to +1: NDC > 0: net shift toward higher categories; NDC < 0: net shift toward lower categories; NDC = 0: no net directional change.

**Table 4:** P-values obtained for the changes in the scores obtained by participants who completed the study across the different dimensions before and after the educational strategy. Santander, Colombia, 2025 (*n* = 58).

Variables	Dimensions		
	D	K	P
	*p*-Value *	*p*-Value *	*p*-Value *
Age categorized			
≤26	0.100	<0.001	<0.001
≥27	0.001	0.001	<0.001
Marital status			
Single	0.103	0.004	0.002
Married/Cohabiting	0.319	<0.001	<0.001
Education			
≤Primary	0.077	0.003	<0.001
Secondary	0.334	<0.001	<0.001
Technical- Technological	0.317	0.066	0.273
Number of children			
One	0.115	<0.001	<0.001
Two and more	0.369	<0.001	<0.001
Occupation			
Employed	0.655	0.414	0.102
Housewife	0.066	<0.001	<0.001
Independent	0.317	0.109	0.285
Student	1.000	0.317	0.317
Socioeconomic stratum			
Low (1–2)	0.096	<0.001	<0.001
Medium (3–4)	0.655	0.180	0.180
Origin Country			
Colombia	0.099	<0.001	<0.001
Venezuela	0.518	0.012	0.012
Ethnicity			
No	0.134	<0.001	<0.001
Indigenous	0.317	0.317	0.317

* Nonparametric Wilcoxon signed-rank test.

## Data Availability

Data is unavailable due to privacy and ethical restrictions.

## Supplementary Materials

The following supporting information can be downloaded at: https://www.mdpi.com/article/10.3390/pediatric18020041/s1; Table S1: Participants’ responses to the questions related to the psychological dimension. Moment 1. Santander, Colombia, 2025 (*n* = 58); Table S2: Participants’ responses to the questions related to the psychological dimension. Moment 2. Santander, Colombia, 2025 (*n* = 58). Table S3: Participants’ responses to the questions related to the oral health knowledge dimension. Moment 1. Santander, Colombia, 2025 (*n* = 58). Table S4: Participants’ responses to the questions related to the oral health knowledge dimension. Moment 2. Santander, Colombia, 2025 (*n* = 58). Table S5: Participants’ responses to the questions related to the oral health practices dimension. Moment 1. Santander, Colombia (*n* = 58). Table S6: Participants’ responses to the questions related to the oral health practices dimension. Moment 2. Santander, Colombia (*n* = 58). Table S7: Bivariate comparisons between sociodemographic characteristics and psychosocial, knowledge, and oral health practice scores at Moment 2. Santander, Colombia, 2025 (*n* = 58). Table S8. Bivariate comparisons between sociodemographic characteristics and excellent levels across dimensions at Moment 2. Santander, Colombia, 2025 (*n* = 58).

## Abbreviations

The following abbreviations are used in this manuscript:

KAP	Knowledge, Attitudes, and Practices
D	Psychosocial dimension
K	Oral Health Knowledge
P	Oral Health Practices
GM	Gross mobility
NDC	Net directional change
CIOMS	Council for International Organizations of Medical Sciences
CEBIC	Committee on Ethics, Bioethics, and Scientific Integrity
E.S.E	Three letter acronym in Spanish for Empresa Social del Estado [State Social Enterprise]
DANE	Four letter acronym in Spanish for Departamento Administrativo Nacional de Estadística [National Administrative Department of Statistics of Colombia]
