# Supplementary material for: The Effect of an Educational Strategy on Mothers’ Knowledge and Practices Regarding Their Children’s Oral Health
Source: Pediatr Rep. 2026 Mar 12;18(2):41. doi: 10.3390/pediatric18020041 (PMC13010658; doi:10.3390/pediatric18020041)
Supplement: Supplementary file 1 [file pediatrrep-18-00041-s001.zip › pediatrrep-4117538-supplementary.pdf]

Table S1. Participants' responses to the questions related to the psychological dimension. Moment 1. Santander, Colombia, 2025 (n = 58).

	Psychosocial dimension (related to attitudes and opinions) Moment 1						Categorization of responses	
	Questions/Statements/Answer options	Strongly disagree *n* (%)	Partially disagree *n* (%)	Neither disagree nor agree (neutral) *n* (%)	Partially agree *n* (%)	Strongly agree *n* (%)	Negative *n* (%)	Positive *n* (%)
D1	Breastfeeding enhances the mother-child bond.	7 (12.1)	0 (0.0)	3 (5.2)	3 (5.2)	45 (77.6)	10 (17.2)	48 (82.8)
D2	Maintaining a child's oral health is the responsibility of the parents	7 (12.1)	0 (0.0)	0 (0.0)	4 (6.9)	47 (81.0)	7 (12.1)	51 (87.9)
	Questions/Statements/Answer options	Completely dissatisfied *n* (%)	Dissatisfied *n* (%)	Somewhat satisfied *n* (%)	Satisfied *n* (%)	Completely Satisfied *n* (%)	Negative *n* (%)	Positive *n* (%)
D3	When feeding my baby with a bottle, I will feel	5 (8.6)	13 (22.4)	16 (27.6)	17 (29.3)	7 (12.1)	18 (31.0)	40 (69.0)
D4	I consider that as a mother, while breastfeeding, I will feel…	2 (3.4)	0 (0.0)	3 (5.2)	13 (22.4)	40 (69.0)	2 (3.4)	56 (96.6)
	Questions/Statements/Answer options	Not useful at all *n* (%)	Not very useful *n* (%)	Somewhat useful *n* (%)	Very useful *n* (%)	Extremely useful *n* (%)	Negative *n* (%)	Positive *n* (%)
D5	Acquiring knowledge about oral care and hygiene practices for my baby is..	0 (0.0)	0 (0.0)	7 (12.1)	25 (43.1)	26 (44.8)	7 (12.1)	51 (87.9)

D6 Learning about how to feed my baby during the first year of life is…	0 (0.0)	0 (0.0)	4 (6.9)	24 (41.4)	30 (51.7)	4 (6.9)	54 (93.1)
Questions/Statements/Answer options	Not important *n* (%)	Slightly important *n* (%)	Somewhat important (neutral) *n* (%)	Important *n* (%)	Very important *n* (%)	Negative *n* (%)	Positive *n* (%)
D7 I consider that breastfeeding my baby is…	0 (0.0)	0 (0.0)	1 (1.7)	18 (31.0)	39 (67.2)	1 (1.7)	57 (98.3)

Table S2. Participants' responses to the questions related to the psychological dimension. Moment 2. Santander, Colombia, 2025 (n = 58).

	**Psychosocial dimension (related to attitudes and opinions) Moment 2**						**Categorization of responses**	
	Questions/Statements/Answer options	Strongly disagree *n* (%)	Partially disagree *n* (%)	Neither disagree nor agree (neutral) *n* (%)	Partially agree *n* (%)	Strongly agree *n* (%)	Negative *n* (%)	Positive *n* (%)
D1	Breastfeeding enhances the mother-child bond.	2 (3.4)	0 (0.0)	0 (0.0)	2 (3.4)	54 (93.1)	2 (3.4)	56 (96.6)
D2	Maintaining a child's oral health is the responsibility of the parents	2 (3.4)	0 (0.0)	0 (0.0)	3 (5.2)	53 (91.4)	2 (3.4)	56 (96.6)
	Questions/Statements/Answer options	Completely dissatisfied *n* (%)	Dissatisfied *n* (%)	Somewhat satisfied *n* (%)	Satisfied *n* (%)	Completely Satisfied *n* (%)	Negative *n* (%)	Positive *n* (%)
D3	When feeding my baby with a bottle, I will feel	3 (5.2)	20 (34.5)	13 (22.4)	16 (27.6)	6 (10.3)	23 (39.7)	35 (60.3)
D4	I consider that as a mother, while breastfeeding, I will feel…	1 (1.7)	1 (1.7)	0 (0.0)	19 (32.8)	37 (63.8)	2 (3.4)	56 (96.6)
	Questions/Statements/Answer options	Not useful at all *n* (%)	Not very useful *n* (%)	Somewhat useful *n* (%)	Very useful *n* (%)	Extremely useful *n* (%)	Negative *n* (%)	Positive *n* (%)
D5	Acquiring knowledge about oral care and hygiene practices for my baby is..	0 (0.0)	0 (0.0)	1 (1.7)	32 (55.2)	25 (43.1)	0 (0.0)	58 (100.0)

D6	Learning about how to feed my baby during the first year of life is…	0 (0.0)	0 (0.0)	1 (1.7)	32 (55.2)	25 (43.1)	0 (0.0)	58 (100.0)

Questions/Statements/Answer options		Not important *n* (%)	Slightly important *n* (%)	Somewhat important (neutral) *n* (%)	Important *n* (%)	Very important *n* (%)	Negative *n* (%)	Positive *n* (%)
D7	I consider that breastfeeding my baby is…	0 (0.0)	0 (0.0)	1 (1.7)	15 (25.9)	42 (72.4)	1 (1.7)	57 (98.3)

Table S3. Participants' responses to the questions related to the oral health knowledge dimension. Moment 1. Santander, Colombia, 2025 (*n* = 58).

Oral health knowledge questions/statements Moment 1		Answer Options (I am…)					Categorization of responses	
		Strongly disagree *n* (%)	Partially disagree *n* (%)	Neither disagree nor agree (neutral) *n* (%)	Partially agree *n* (%)	Strongly agree *n* (%)	Incorrect *n* (%)	Correct *n* (%)
K1	Formula milk (canned milk) currently used is as nutritious as breast milk	22 (37.9)	13 (22.4)	16 (27.6)	4 (6.9)	3 (5.2)	23 (39.7)	35 (60.3)
K2	Breast milk has greater benefits than formula feeding	3 (5.2)	0 (0.0)	4 (6.9)	14 (24.1)	37 (63.8)	7 (12.1)	51 (87.9)
K3	I believe that the mother naturally and instinctively learns how to breastfeed her baby	3 (5.2)	3 (5.2)	7 (12.1)	26 (44.8)	19 (32.8)	13 (22.4)	45 (77.6)
K4	Babies who are breastfed are healthier than those who are fed with formula	1 (1.7)	1 (1.7)	16 (27.6)	16 (27.6)	24 (41.4)	18 (31.0)	40 (69.0)
K5	Feeding my child at night with formula milk or a beverage containing sugar affects their teeth	4 (6.9)	2 (3.4)	21 (36.2)	17 (29.3)	14 (24.1)	27 (46.6)	31 (53.4)
K6	Breastfeeding becomes difficult when the mother works outside the home	5 (8.6)	1 (1.7)	24 (41.4)	12 (20.7)	16 (27.6)	18 (31.0)	40 (69.0)
K7	The child's oral health is related to their general health	4 (6.9)	0 (0.0)	12 (20.7)	24 (41.4)	16 (27.6)	16 (27.6)	42 (72.4)
K8	Stains on the surfaces of the teeth are the first signs of dental caries	5 (8.6)	1 (1.7)	24 (41.4)	12 (20.7)	16 (27.6)	30 (51.7)	28 (48.3)

K9	Dental cavities are caused by bacteria that are transmitted when feeding utensils are shared (i.e spoons)	8 (13.8)	10 (17.2)	19 (32.8)	10 (17.2)	11 (19.0)	37 (63.8)	21 (36.2)
K10	Toothpaste with fluoride will help prevent dental cavities in my child	3 (5.2)	6 (10.3)	30 (51.7)	11 (19.0)	8 (13.8)	39 (67.2)	18 (31.0)
K11	The mother's diet during pregnancy affects the development of the baby's teeth	10 (17.2)	7 (12.1)	23 (39.7)	10 (17.2)	8 (13.8)	40 (69.0)	18 (31.0)
K12	Breastfeeding my child will help with better growth of the face and teeth	4 (6.9)	8 (13.8)	15 (25.9)	12 (20.7)	19 (32.8)	27 (46.6)	31 (53.4)
K13	Habits (lip sucking, finger sucking, cloth sucking, and use of pacifiers) will affect the development of teeth and facial bones	5 (8.6)	5 (8.6)	17 (29.3)	14 (24.1)	17 (29.3)	27 (46.6)	31 (53.4)

Table S4. Participants' responses to the questions related to the oral health knowledge dimension. Moment 2. Santander, Colombia, 2025 (n = 58).

Oral health knowledge questions/statements Moment 2		Answer Options (I am…)					Categorization of responses	
		Strongly disagree *n* (%)	Partially disagree *n* (%)	Neither disagree nor agree (neutral) *n* (%)	Partially agree *n* (%)	Strongly agree *n* (%)	Incorrect *n* (%)	Correct *n* (%)
K1	Formula milk (canned milk) currently used is as nutritious as breast milk	24 (41.4)	17 (29.3)	10 (17.2)	5 (8.6)	2 (3.4)	17 (29.3)	41 (70.7)
K2	Breast milk has greater benefits than formula feeding	0 (0.0)	0 (0.0)	2 (3.4)	2 (3.4)	54 (93.1)	2 (3.4)	56 (96.6)
K3	I believe that the mother naturally and instinctively learns how to breastfeed her baby	2 (3.4)	0 (0.0)	5 (8.6)	25 (43.1)	26 (44.8)	7 (12.1)	51 (87.9)
K4	Babies who are breastfed are healthier than those who are fed with formula	0 (0.0)	1 (1.7)	4 (6.9)	12 (20.7)	41 (70.7)	5 (8.6)	53 (91.4)
K5	Feeding my child at night with formula milk or a beverage containing sugar affects their teeth	0 (0.0)	2 (3.4)	11 (19.0)	14 (24.1)	31 (53.4)	13 (22.4)	45 (77.6)
K6	Breastfeeding becomes difficult when the mother works outside the home	0 (0.0)	1 (1.7)	6 (10.3)	25 (43.1)	26 (44.8)	7 (12.1)	51 (87.9)
K7	The child's oral health is related to their general health	1 (1.7)	1 (1.7)	3 (5.2)	23 (39.7)	30 (51.7)	5 (8.6)	53 (91.4)
K8	Stains on the surfaces of the teeth are the first signs of dental caries	0 (0.0)	0 (0.0)	9 (15.5)	29 (50.0)	20 (34.5)	9 (15.5)	49 (84.5)

K9	Dental cavities are caused by bacteria that are transmitted when feeding utensils are shared (i.e spoons)	0 (0.0)	0 (0.0)	10 (17.2)	29 (50.0)	19 (32.8)	10 (17.2)	48 (82.8)
K10	Toothpaste with fluoride will help prevent dental cavities in my child	0 (0.0)	1 (1.7)	9 (15.5)	26 (44.8)	22 (37.9)	10 (17.2)	48 (82.8)
K11	The mother's diet during pregnancy affects the development of the baby's teeth	4 (6.9)	0 (0.0)	8 (13.8)	30 (51.7)	16 (27.6)	12 (20.7)	46 (79.3)
K12	Breastfeeding my child will help with better growth of the face and teeth	0 (0.0)	2 (3.4)	5 (8.6)	31 (53.4)	20 (34.5)	7 (12.1)	51 (87.9)
K13	Habits (lip sucking, finger sucking, cloth sucking, and use of pacifiers) will affect the development of teeth and facial bones	0 (0.0)	1 (1.7)	1 (1.7)	22 (37.9)	34 (58.6)	2 (3.4)	56 (96.6)

Table S5. Participants' responses to the questions related to the oral health practices dimension. Moment 1. Santander, Colombia (n = 58)

<table>
<thead>
<tr><th rowspan="2"></th><th rowspan="2">Oral health practices questions/statements Moment 1</th><th colspan="6" rowspan="2">Answer options</th><th colspan="2">Categorization of responses</th></tr>
<tr><th>Inadequate n (%)</th><th>Adequate n (%)</th></tr>
</thead>
<tbody>
<tr><td rowspan="2">P1</td><td rowspan="2">How often will you breastfeed your baby in the first six months of age?</td><td colspan="2">Every two hours n (%)</td><td colspan="2">When the baby is awake n (%)</td><td colspan="2">When the baby desires it n (%)</td><td rowspan="2">26 (44.8)</td><td rowspan="2">32 (55.2)</td></tr>
<tr><td colspan="2">19 (32.8)</td><td colspan="2">7 (12.1)</td><td colspan="2">32 (55.2)</td></tr>
<tr><td rowspan="2">P2</td><td rowspan="2">If you work outside the home, how will you feed the baby?</td><td colspan="2">I would have to give him/her formula milk n (%)</td><td colspan="2">Breastfeeding when I'm with the baby and formula milk when I'm not with him/her n (%)</td><td colspan="2">I would give breast milk expressed at home or work n (%)</td><td rowspan="2">23 (39.7)</td><td rowspan="2">46 (79.3)</td></tr>
<tr><td colspan="2">4 (6.9)</td><td colspan="2">19 (32.8)</td><td colspan="2">35 (60.3)</td></tr>
<tr><td rowspan="2">P3</td><td rowspan="2">Regarding baby teeth and permanent teeth, I think that:</td><td colspan="2">Baby teeth are not important at all n (%)</td><td colspan="2">Baby teeth are slightly important n (%)</td><td colspan="2">Baby teeth and permanent teeth are equally important n (%)</td><td rowspan="2">12 (20.7)</td><td rowspan="2">46 (79.3)</td></tr>
<tr><td colspan="2">2 (3.4)</td><td colspan="2">10 (17.2)</td><td colspan="2">46 (79.3)</td></tr>
<tr><td rowspan="2">P4</td><td rowspan="2">Until what age will you breastfeed your baby?</td><td>Three months n (%)</td><td>Six months n (%)</td><td>One year n (%)</td><td>Two years n (%)</td><td>Until the baby wants to n (%)</td><td>Other n (%)</td><td rowspan="2">42 (72.4)</td><td rowspan="2">16 (27.6)</td></tr>
<tr><td>2 (3.4)</td><td>9 (15.5)</td><td>23 (39.7)</td><td>16 (27.6)</td><td>8 (13.8)</td><td>0 (0.0)</td></tr>
<tr><td rowspan="2">P5</td><td rowspan="2">Who would carry out the baby's oral care practices?</td><td colspan="2">Yourself n (%)</td><td>A family member n (%)</td><td colspan="2">External caregiver n (%)</td><td>Other n (%)</td><td rowspan="2">7 (12.1)</td><td rowspan="2">51 (87.9)</td></tr>
<tr><td colspan="2">51 (87.9)</td><td>4 (6.9)</td><td colspan="2">1 (1.7)</td><td>2 (3.4)</td></tr>
</tbody>
</table>

P6	How will you feed your baby until six months?	Only with breast milk *n* (%)	Primarily with formula milk *n* (%)	Breastfeeding and formula milk *n* (%)	Other n (%)		14 (24.1)	44 (75.9)
		44 (75.9)	3 (5.2)	11 (19.0)	0 (0.0)			
P7	How will you feed your child from six months to one year of age?	Only with breast milk *n* (%)	Breastfeeding and formula milk *n* (%)	Breastfeeding plus complementary feeding *n* (%)	Only complementary feeding *n* (%)		13 (22.4)	45 (77.6)
		7 (12.1)	3 (5.2)	45 (77.6)	3 (5.2)			
P8	At what age will you take your child for their first visit to the dentist?	At birth *n* (%)	Between six months and one year of age *n* (%)	After six years of age *n* (%)	When the child feels any pain *n* (%)		9 (15.5)	49 (84.5)
		1 (1.7)	49 (84.5)	6 (10.3)	2 (3.4)			
P9	When do you think the cleaning of the baby's mouth should begin?	As soon as the first tooth appears *n* (%)	After the eruption of all the primary teeth *n* (%)	The gums should be cleaned regularly after birth *n* (%)	I do not know *n* (%)		38 (65.5)	20 (34.5)
		26 (44.8)	2 (3.4)	20 (34.5)	10 (17.2)			
P10	How often should you clean the baby's mouth?	Once a day *n* (%)	Regularly after each feeding *n* (%)	No cleaning is required before the teeth appear *n* (%)	I do not know *n* (%)		49 (84.5)	9 (15.5)
		9 (15.5)	33 (56.9)	5 (8.6)	11 (19.0)			
P11	How will you clean your baby's mouth before the teeth come in?	With water *n* (%)	With a wet gauze *n* (%)	With a toothbrush *n* (%)	With toothpaste *n* (%)	I should not *n* (%)	18 (31.0)	40 (69.0)
		3 (5.2)	40 (69.0)	4 (6.9)	0 (0.0)	11 (19.0)		

Table S6. Participants' responses to the questions related to the oral health practices dimension. Moment 2. Santander, Colombia (*n* = 58)

	**Oral health practices questions/statements Moment 2**	**Answer options**						**Categorization of responses**	
								**Inadequate n (%)**	**Adequate n (%)**
P1	How often will you breastfeed your baby in the first six months of age?	Every two hours *n* (%)	When the baby is awake *n* (%)	When the baby desires it *n* (%)				10 (17.2)	48 (82.8)
		8 (13.8)	2 (3.4)	48 (82.8)					
P2	If you work outside the home, how will you feed the baby?	I would have to give him/her formula milk *n* (%)	Breastfeeding when I'm with the baby and formula milk when I'm not with him/her *n* (%)	I would give breast milk expressed at home or work n (%)				9 (15.5)	49 (84.5)
		1 (1.7)	8 (13.8)	49 (84.5)					
P3	Regarding baby teeth and permanent teeth, I think that:	Baby teeth are not important at all *n* (%)	Baby teeth are slightly important *n* (%)	Baby teeth and permanent teeth are equally important *n* (%)				5 (6.9)	53 (91.4)
		4 (6.9)	1 (1.7)	53 (91.4)					
P4	Until what age will you breastfeed your baby?	Three months *n* (%)	Six months *n* (%)	One year *n* (%)	Two years *n* (%)	Until the baby wants to *n* (%)	Other *n* (%)	19 (32.8)	39 (67.2)
		1 (1.7)	2 (3.4)	12 (20.7)	39 (67.2)	4 (6.9)	0 (0.0)		
P5	Who would carry out the baby's oral care practices?	Yourself *n* (%)	A family member n (%)	External caregiver *n* (%)	Other *n* (%)			1 (1.7)	57 (98.3)
		57 (98.3)	1 (1.7)	0 (0.0)	0 (0.0)				
P6	How will you feed your baby until six months?	Only with breast milk n (%)	Primarily with formula milk n (%)	Breastfeeding and formula milk n (%)	Other n (%)			4 (6.9)	54 (93.1)

		54 (93.1)	1 (1.7)	3 (5.2)		0 (0.0)		
P7	How will you feed your child from six months to one year of age?	Only with breast milk<br>*n* (%)	Breastfeeding and formula milk<br>*n* (%)	Breastfeeding plus complementary feeding<br>*n* (%)		Only complementary feeding<br>*n* (%)	5 (8.6)	53 (91.4)
		1 (1.7)	4 (6.9)	53 (91.4)		0 (0.0)		
P8	At what age will you take your child for their first visit to the dentist?	At birth<br>*n* (%)	Between six months and one year of age<br>*n* (%)	After six years of age<br>*n* (%)		When the child feels any pain<br>*n* (%)	9 (15.5)	49 (84.5)
		0 (0.0)	49 (84.5)	9 (15.5)		0 (0.0)		
P9	When do you think the cleaning of the baby’s mouth should begin?	As soon as the first tooth appears<br>*n* (%)	After the eruption of all the primary teeth<br>*n* (%)	The gums should be cleaned regularly after birth<br>*n* (%)		I do not know<br>*n* (%)	12 (20.7)	46 (79.3)
		7 (12.1)	3 (5.2)	46 (79.3)		2 (3.4)		
P10	How often should you clean the baby's mouth?	Once a day<br>*n* (%)	Regularly after each feeding<br>*n* (%)	No cleaning is required before the teeth appear<br>*n* (%)		I do not know<br>*n* (%)	16 (27.6)	42 (72.4)
		15 (25.9)	42 (72.4)	1 (1.7)		0 (0.0)		
P11	How will you clean your baby's mouth before the teeth come in?	With water<br>*n* (%)	With a wet gauze<br>*n* (%)	With a toothbrush<br>*n* (%)	With toothbrush/toothpaste<br>*n* (%)	I should not<br>*n* (%)	4 (6.9)	54 (93.1)
		2 (3.4)	2 (3.4)	38 (65.5)	16 (27.6)	0 (0.0)		

Table S7. Bivariate comparisons between sociodemographic characteristics and psychosocial, knowledge, and oral health practice scores at Moment 2. Santander, Colombia, 2025 (n = 58)

Variables	Psychosocial (score)		Knowledge (score)		Practices (score)		Knowledge and practices (score)		All dimensions (score)	
	Median (IQR)	p-value*	Median (IQR)	p-value*	Median (IQR)	p-value*	Median (IQR)	p-value*	Median (IQR)	p-value*
Age categorized										
≤ 26	100.0 (14.3)	0.542	92.3 (23.1)	0.383	90.9 (18.8)	0.838	91.7 (16.6)	0.568	90.3 (12.9)	0.665
≥ 27	100.0 (14.3)		84.6 (23.1)		81.2 (27.3)		83.3 (20.8)		87.1 (12.9)	
Marital status										
Single	85.7 (14.3)	0.239	92.3 (19.2)	0.355	86.4 (9.1)	0.906	91.7 (13.5)	0.734	90.3 (13.8)	0.816
Married/Cohabiting	100.0 (14.3)		88.4 (23.1)		90.9 (27.3)		85.4 (20.8)		87.1 (12.9)	
Education										
≤ Primary	100.0 (14.3)	0.362	79.9 (23.1)	0.044	81.2 (18.2)	0.025	79.2 (20.8)	0.203	83.9 (16.1)	0.290
Secondary	85.7 (14.3)		93.3 (15.4)		90.9 (18.2)		91.7 (16.6)		90.3 (12.9)	
Technical- Technological	100.0 (14.3)		100.0 (11.5)		68.2 (15.9)		83.3 (9.4)		87.1 (9.7)	
Number of children										
One	92.9 (14.3)	0.415	92.3 (23.1)	0.700	90.9 (18.2)	0.450	91.7 (15.6)	0.813	90.3 (12.1)	0.994
Two and more	100.0 (14.3)		88.5 (23.1)		86.4 (20.5)		85.4 (20.8)		85.5 (17.0)	
Occupation										
Employed	78.6 (--)	0.140	80.8 (--)	0.296	81.2 (--)	0.302	81.3 (16.6)	0.380	80.7 (--)	0.228
Housewife	100.0 (14.3)		92.3 (23.1)		86.4 (27.3)		89.6 (16.6)		90.3 (12.9)	
Independent	100.0 (14.3)		100.0 (15.4)		90.9 (18.2)		91.7 (12.5)		93.5 (9.7)	
Student	---		---		---		---		---	
Socioeconomic stratum										
Low (1-2)	100.0 (14.3)	0.068	92.3 (23.1)	0.559	90.9 (27.3)	0.640	91.7 (19.8)	0.640	90.3 (12.9)	0.350
Medium (3-4)	78.5 (14.3)		---		81.8 (18.2)		83.4 (--)		82.3 (---)	
Origin Country										

Colombia	100.0 (14.3)	0.650	92.3 (23.1)	0.587	86.4 (27.3)	0.682	91.7 (17.6)	0.851	90.3 (12.9)	0.834
Venezuela	92.9 (14.3)		84.6 (25.0)		90.9 (22.7)		87.5 (18.7)		85.5 (15.4)	
Ethnicity										
No	100.0 (14.3)	0.552	92.3 (23.1)	0.034	90.9 (27.3)	0.793	91.7 (16.6)	0.034	90.3 (12.9)	0.034
Indigenous	--		--		--		--		--	

IQR- Interquartile range; * No parametric tests were used: Mann-Whitney U test for dichotomous variables, Kruskal-Wallis test for polychotomous variables.

Table S8. Bivariate comparisons between sociodemographic characteristics and excellent levels across dimensions at Moment 2. Santander, Colombia, 2025 (n = 58)

Variables	Psychosocial (excellent)		Knowledge (excellent)		Practices (excellent)		Knowledge and practices (excellent)		All dimensions (excellent)	
	n (%)	p-value*	*n* (%)	p-value*	*n* (%)	p-value*	*n* (%)	p-value*	*n* (%)	p-value*
Age categorized										
≤ 26	34 (97.1)	1.000	26 (74.3)	0.655	26 (74.3)	0.458	22 (62.9)	0.835	29 (82.9)	0.925
≥ 27	22 (95.7)		15 (65.2)		15 (65.2)		13 (56.5)		18 (78.3)	
Marital status										
Single	11 (91.7)	0.878	9 (75.0)	0.713	10 (83.3)	0.469	9 (75.0)	0.404	10 (83.3)	1.000
Married/Cohabiting	45 (97.8)		32 (69.6)		31 (67.4)		26 (56.5)		37 (80.4)	
Education										
≤ Primary	19 (100.0)	0.506	9 (47.4)	0.017	12 (63.2)	0.049	7 (36.8)	0.038	14 (73.7)	0.432
Secondary	33 (94.3)		28 (80.0)		28 (80.0)		25 (71.4)		29 (82.9)	
Technical- Technological	4 (100.0)		4 (100.0)		1 (25.0)		3 (75.0)		4 (100.0)	
Number of children										
One	31 (96.9)	0.881	23 (71.9)	1.000	25 (78.1)	0.276	20 (62.5)	0.918	27 (84.4)	0.702
Two and more	25 (96.2)		18 (69.2)		16 (61.5)		15 (57.7)		20 (76.9)	
Occupation										
Employed	1 (50.0)	0.004	1 (50.0)	0.642	1 (50.0)	0.310	1 (50.0)	0.311	1 (50.0)	0.067
Housewife	47 (97.9)		33 (68.8)		34 (70.8)		28 (58.3)		39 (81.3)	
Independent	0 (0.0)		6 (85.7)		6 (85.7)		6 (85.7)		7 (100.0)	
Student	0 (0.0)		1 (100.0)		0 (0.0)		0 (0.0)		0 (0.0)	
Socioeconomic stratum										
Low (1-2)	55 (98.2)	0.089	39 (69.6)	0.892	40 (71.4)	1.000	34 (60.7)	1.000	45 (80.4)	1.000
Medium (3-4)	1 (50.0)		2 (100.0)		1 (50.0)		1 (50.0)		2 (100.0)	
Origin Country										
Colombia	49 (98.0)	0.640	35 (70.0)	1.000	35 (70.0)	1.000	30 (60.0)	1.000	40 (80.0)	0.987

Venezuela	7 (87.5)		6 (75.0)		6 (75.0)		5 (62.5)		7 (87.5)	
Ethnicity										
No	55 (96.5)	1.000	41 (71.9)	0.647	40 (70.2)	1.000	35 (61.4)	0.831	0 (0.0)	0.425
Indigenous	1 (100.0)		0 (0.0)		1 (100.0)		0 (0.0)		47 (82.5)	
Total	56 (96.6)	---	41 (70.7)	---	41 (70.7)	---	35 (60.3)	---	47 (81.0)	---

* Chi-square test with Yates' continuity correction or Fisher's exact test, as appropriate.
